# Handling and Associated Material Properties of Porcine and Human Acellular Dermal Matrices

**DOI:** 10.1093/asjof/ojaf083

**Published:** 2025-07-02

**Authors:** Allen Gabriel, Rafael Gottenger, Nimesh Kabaria, Patrick Leamy, Maryellen Gardocki-Sandor, Erin M Black

## Abstract

**Background:**

Pliability, handling, and drape characteristics are important material properties of acellular dermal matrices (ADMs). There are few in vitro techniques that quantitatively assess these characteristics.

**Objectives:**

The aim of the authors of this study is to compare data generated using a novel drape assay with surgeon handling of porcine-derived ADMs and evaluate material properties of human- and porcine-derived ADMs.

**Methods:**

Three commercially available ADMs (human-derived AlloDerm [hADM], porcine-derived Strattice Pliable [pADM-S], and porcine-derived Artia [pADM-A]) were assessed. Eight prototype variations of pADM with varied mechanical properties were used for the handling assessment. Drape testing was conducted using a novel mechanical test fixture coupled with quantitative image analysis. Surgeon handling assessments of prototype pADM pliability were compared with quantitative drape measurements to establish a correlation. Commercially available ADMs were tensile tested mechanically at clinically relevant and higher loads to determine maximum strength.

**Results:**

Benchtop testing demonstrated similar drapability for pADM-A and hADM; pADM-S had significantly less drapability than pADM-A, despite both being porcine derived. Surgeon semiquantitative ranking of pliability of prototype pADM samples positively correlated with drape measurements (*R*^2^ = 82.5%, *P* = .000). Tensile testing demonstrated that hADM had the greatest degree of strain/elongation across all applied loads, followed by pADM-A, then pADM-S. When tested to failure, hADM had the greatest average maximum stress compared with pADM-A and pADM-S.

**Conclusions:**

The novel drape assay introduced here correlated with surgeon handling assessment of ADM pliability. Material testing and handling assessment demonstrated various mechanical properties (ie, strength, strain/elongation, drapability, and handling/pliability) for the ADMs evaluated, providing several unique options that may meet different surgical mechanical needs.

**Level of Evidence: 5 (Therapeutic):**

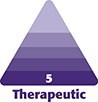

Acellular dermal matrices (ADMs) are commonly used in both plastic and reconstructive surgical procedures to provide soft tissue reinforcement and support across large and small defects.^1-3^ Some studies demonstrate that the use of ADMs in surgical procedures can reduce rates of certain complications, such as seroma and infection, compared with procedures that do not use ADM.^1,3^

ADMs are most commonly derived from human, porcine, or bovine dermis.^2,3^ Following harvest of native tissue, the tissues are subjected to processing procedures to create the ADM, which includes removal of the epithelium and any cellular components within the dermis that may lead to an immunogenic inflammatory reaction.^2,3^

Processing and ADM sterilization procedures can involve chemical, biochemical, or physical techniques, or a combination thereof, which can result in ADMs with different material properties and can lead to varying host responses following implantation.^4^ Similarly, the origin of the ADM tissues can also influence the ADM material properties.^4^

A key property to maintain soft tissue coverage and improve aesthetic outcomes with ADMs is pliability or flexibility in terms of surgeon handling, with the property of drapability, or the ability of an ADM to conform to a surface, being closely related.^2^ Depending on the native species of origin and postharvesting procedures, ADMs can have varying degrees of pliability. Although several studies have demonstrated comparisons of ultimate strength and material properties of various ADMs, there are few techniques available to quantitatively assess the pliability or drapability of ADMs in a laboratory setting to determine the most appropriate choice for a specific application in plastic and reconstructive surgery.^5-7^

The objective of these studies was to evaluate ADMs derived from human or porcine tissue to determine raw material and processing procedure effects on material properties. Additionally, the correlation of a novel drape assay with surgeon handling of porcine-derived ADMs was assessed.

## METHODS

### Materials Assessed

ADMs used in these studies included 3 commercially available ADMs and 8 variations of a porcine-derived prototype ADM. The commercially available ADMs (all Allergan Aesthetics, an AbbVie Company, Branchburg, NJ) included a human-derived ADM (hADM [AlloDerm]) and 2 porcine-derived ADMs (pADM-S [Strattice Pliable] and pADM-A [Artia]). Eight variations of the prototype pADM were created for the purpose of semiquantitative handling assessment.

### Drape Measurements

**Figure 1.**
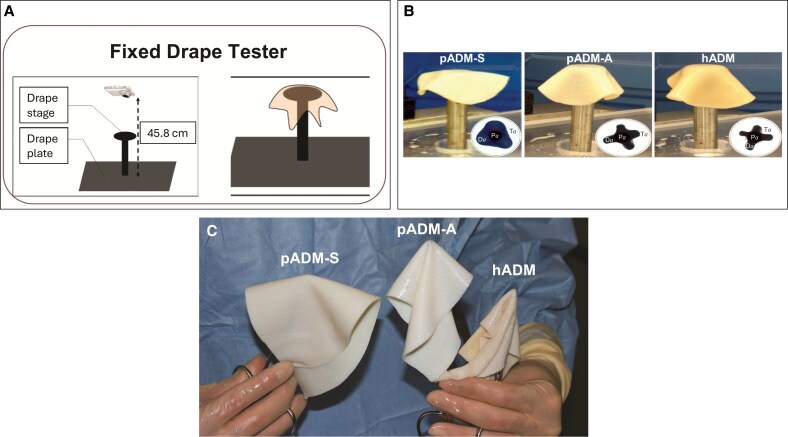
Material properties assessment. (A) Diagram of a fixed drape tester, (B) representative images of draped commercial ADMs, and (C) representative images of commercial ADMs during drape testing. ADM, acellular dermal matrix; Da, draped tissue surface area; hADM, human-derived ADM; N, Newton; Pa, platform surface area; pADM-A, porcine-derived ADM (Artia), pADM-S, porcine-derived ADM (Strattice Pliable); Ta, undraped tissue surface area.

A novel quantitative material drape assessment assay was developed to assess surgeon handling of commercially available ADMs. This test relies on the change in ratio of draped vs undraped surface area, which is determined using an image analysis software tool (ImageJ; National Institutes of Health, Bethesda, MD). Drape testing involved lowering a 6-cm disk-shaped piece of ADM onto a fixed pedestal and allowing it to drape in place for 2 s (Figure 1A). Change in perceived 2-dimensional surface area was then measured (Figure 1B). Outputs from drape testing were quantified in drape units, with a low value indicating high pliability and a high value indicating greater stiffness or lack of pliability:


$$\mathrm{Drape}=\frac{\mathrm{Da}-\mathrm{Pa}}{\mathrm{Ta}-\mathrm{Pa}}$$

where Da is the draped tissue surface area, Pa is the platform surface area, and Ta is the undraped tissue surface area.

### Handling Assessment

Surgeons (*n* = 23) assessed the handling of 8 pADM prototypes. All of the pADM prototypes had a target thickness of 1 ± 0.25 mm, and they varied in the degree of pliability. Surgeons in the United States who routinely perform plastic and reconstructive procedures and have experience using ADMs were asked to rank the samples in order of handling, from most pliable to least pliable. Outcomes of the surgeon handling and pliability assessments were compared with quantitative drape measurements to establish a correlation between surgeon handling preference and ADM drape measurements.

Instron materials tester and snap gauge measurements were used to determine elongation of the ADMs at clinically relevant loads and sample thicknesses. Tensile testing assessed clinically relevant degrees of strain at 0.5-, 1.5-, and 5.0-newton (N) loads per 2-cm-wide specimens that were normalized by sample thickness. Tensile testing at higher loads determined the maximum stress that can be applied to the ADM before failure.

## RESULTS

### Drape Testing and Correlation With Surgeon Handling Assessment

**Figure 2.**
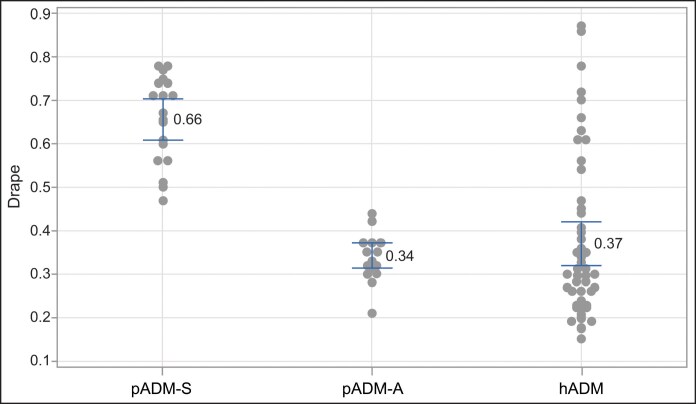
Quantitative drape measurements. Drape testing of commercial ADMs, pADM-S, pADM-A, and hADM. Individual standard deviations were used to calculate the intervals. Data are 95% CIs for the mean. ADM, acellular dermal matrix; hADM, human-derived ADM; pADM-A, porcine-derived ADM (Artia); p-ADM-S, porcine-derived ADM (Strattice Pliable).

Drape testing demonstrated pADM-A had similar properties to hADM, whereas pADM-S had significantly less drapability than pADM-A, despite both being porcine derived (Figures 1C, 2).

**Figure 3.**
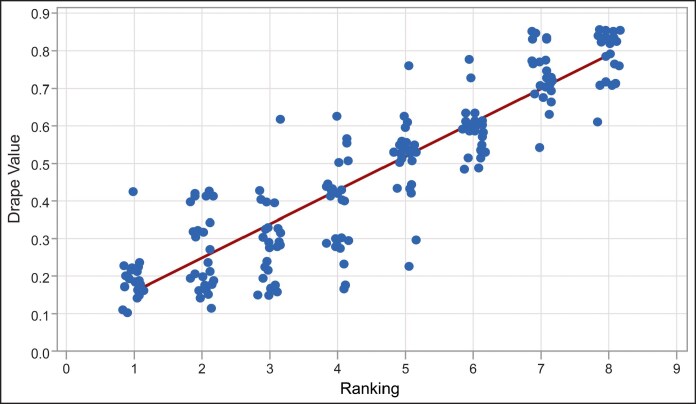
Correlation between quantitative drape testing and semiquantitative surgeon handling assessment. Surgeon handling assessment of pliability for various pADM prototype samples was correlated with the quantitative drape testing measurements. pADM, porcine-derived acellular dermal matrix. The regression equation is 0.06917 + 0.08955 ranking; *P* = .000; *R*^2^ = 82.5%.

The semiquantitative surgeon handling assessment ranking of pliability for the 8 prototype variations of pADM positively correlated with the quantitative drape measurement for those samples (linear regression fitting: *R*^2^ = 82.5%; Pearson's correlation, *P* = .000; Figure 3).

### Mechanical Property Testing

**Figure 4.**
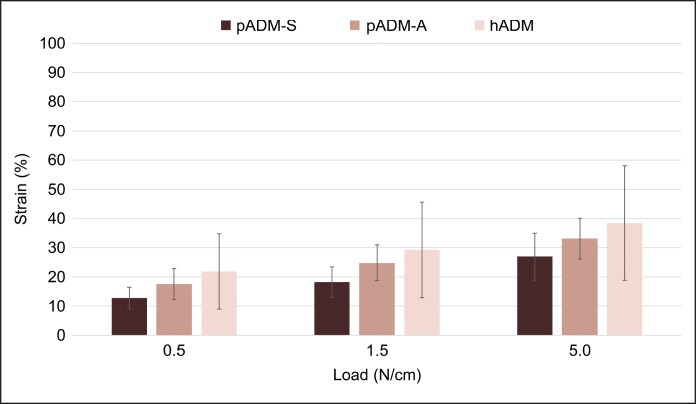
Tensile strain. Strain values for commercial ADMs tested at clinically relevant applied loads. ADM, acellular dermal matrix; hADM, human-derived ADM; N, Newton; pADM-A, porcine-derived ADM (Artia); p-ADM-S, porcine-derived ADM (Strattice Pliable).

The mean (standard deviation [SD]) thicknesses for hADM, pADM-A, and pADM-S were 1.83 (0.83), 1.30 (0.27), and 1.58 (0.24) mm, respectively, with pADM-A being significantly thinner than either hADM (*P* < .05) or pADM-S (*P* < .05). Tensile testing at clinically relevant applied loads of 0.5 to 5.0 N revealed differences in strain for all 3 commercial products. At an applied load of 0.5 N, mean (SD) percent strain was 21.84 (12.9), 17.56 (5.36), and 12.73 (3.72) for hADM, pADM-A, and pADM-S, respectively. At 1.5 N, mean (SD) percent strain was 29.23 (16.38), 24.83 (6.1), and 18.23 (5.22) for hADM, pADM-A, and pADM-S, respectively. At 5 N, mean (SD) percent strain was 38.42 (19.61), 33.09 (6.96), and 26.91 (8.01) for the 3 ADMs assessed, respectively. Overall, hADM exhibited the greatest strain for all 3 applied loads, followed by pADM-A, and finally pADM-S. Additionally, hADM had the greatest variability in strain for all applied loads, followed by pADM-A, then pADM-S (Figure 4).

**Figure 5.**
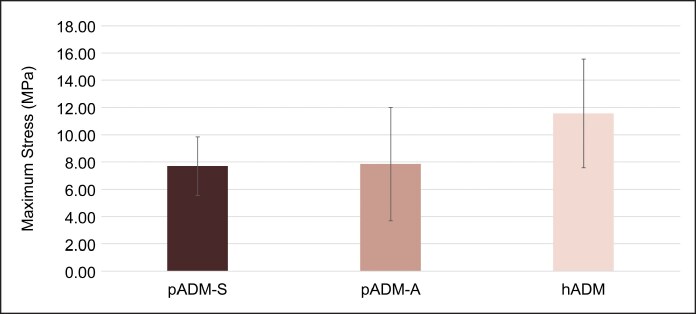
Maximum stress testing. Average maximum stress (±SD) of commercial ADMs, pADM-S, pADM-A, and hADM. ADM, acellular dermal matrix; hADM, human-derived ADM; MPa, MegaPascal; pADM-A, porcine-derived ADM (Artia); p-ADM-S, porcine-derived ADM (Strattice Pliable); SD, standard deviation.

When tensile tested to failure, mean (SD) maximum stress (MPa) was 11.56 (3.99), 7.85 (4.16), and 7.70 (2.14) for hADM, pADM-A, and pADM-S, respectively. hADM had the highest average maximum stress value compared with pADM-A and pADM-S (Figure 5). Stress testing also indicated that pADM-A and pADM-S exhibited similar average maximum stress values.

## DISCUSSION

In this study, the authors evaluated a novel drape assay to quantitatively measure the pliability of ADMs in a laboratory setting in comparison with semiquantitative surgeon handling assessment. Material properties of commercially available ADMs were also assessed at maximum load failure, as well as at clinically relevant subfailure loads.

Surgeon ranking of 8 prototype variations of pADM for tactile assessment of pliability positively correlated with the drape measurements of these samples. This positive correlation with pADM prototype surgeon rankings indicates that the novel drape assay may serve as a predictive tool to assess pliability and handling of ADMs for use in plastic and reconstructive surgery. Drape testing indicated that hADM and pADM-A are equivalently pliable, with both significantly more pliable, and likely more suitable for certain plastic and reconstructive applications, than pADM-S. The ability to assess the pliability and elasticity of ADMs in vitro could provide insight into potential clinical applications of ADMs because these properties may impart suitable mechanical requirements and have been shown to influence the behaviors of infiltrating cells following implantation.^8^

Overall, hADM, pADM-A, and pADM-S all demonstrated different material properties (ie, strength, strain/elongation, pliability, and drapability) for general use in surgical applications, despite the fact that pADM-A and pADM-S are derived from identical tissue sources but processed slightly differently. Tensile testing evaluated the elongation of the ADMs and demonstrated that hADM had the highest and most variable strain/elongation at each of the loads tested, whereas pADM-S exhibited the lowest; pADM-A demonstrated an intermediate strain/elongation between hADM and pADM-S. pADM-A exhibited the lowest variability in strain/elongation compared with the other ADMs tested and had greater strain/elongation than pADM-S. Because of the greater consistency of porcine source material used in the production of pADM-A and pADM-S, the variability of strain values at each applied load was less for both pADM materials compared with hADM, which is produced using human cadaveric donor-derived tissue.^9^ Taken together, these observations demonstrate the range of mechanical and material properties among the 3 ADMs and suggest that pADM-A may have more reliable and predictable deformation than pADM-S and hADM in surgical applications, potentially minimizing variability in outcomes.

The results presented herein indicate that the native tissue source and processing of the ADM may influence the maximum stress level an ADM can tolerate and the variability of stress results. When tested to failure, hADM was able to support the highest maximum stress, with pADM-S and pADM-A tolerating a lower, but similar, level of stress compared with each other. These results are consistent with previous studies demonstrating differences in thickness, maximum load, and/or maximum stress between ADMs from different native sources.^5-7^ Thickness predictably showed a clear impact on overall tensile strength of the ADM samples tested in this study, with pADM-A and pADM-S having more samples with similar thicknesses and similar resulting tensile strengths, and thicker hADM samples demonstrating a greater overall tensile strength. Interestingly, thickness did not appear to have a predictable impact on the other evaluated properties, with hADM exhibiting the greatest strain/elongation, pliability, and drapability. Although pADM-S and pADM-A undergo somewhat different decellularization processes, both ADMs retained equivalent tensile strength while demonstrating significantly different degrees of drapability. This finding allows surgeons a choice in handling and subfailure properties while not compromising strength and durability.

The FDA-cleared clinical use for pADM-S and pADM-A is plastic and reconstructive surgery; for hADM, it is repair and replacement of damaged or inadequate integumental tissue.^10-12^ The differences in material and mechanical properties among the 3 ADMs studied here are subject to surgeon preference and the unique requirements of each surgical case, including the need for greater drapability or pliability, need for greater or lesser elongation based on the size of the defect, need for retained strength because of the demands of the procedure and patient size, need for consistency, and/or the preference for human vs porcine materials.

There are limitations that should be considered when interpreting the results of the current study. The surgeon handling assessment was semiquantitative and, although it was positively correlated with the quantitative drape measurements, caution should be used when determining the clinical relevance of these findings. Because these assessments rely on subjective reports from surgeons, there is the potential for bias, as surgeons’ experiences can vary widely. Additionally, these were benchtop studies to examine the material properties of 3 ADMs. Future in vivo and clinical studies using objective, quantitative assessments with long-term outcomes that evaluate the role of ADMs in tissue repair and various cell populations are warranted.

## CONCLUSIONS

A novel method of evaluating the drape characteristics of commercially available ADMs is presented here. The drapeability of ADMs assessed with this novel method correlated well with the surgeon’s handling assessment of these ADMs for use in surgical applications that require pliability. Results of the material properties testing indicated that hADM exhibited the greatest tensile strain and tolerated the highest maximum stress; pADM-A demonstrated intermediate tensile strain and maximum stress properties between hADM and pADM-S. Mechanical property testing and handling assessment in this study demonstrated various properties for the ADMs evaluated, providing several unique options that may potentially meet different surgical mechanical needs. The clinical trial data associated with this article can be requested online by qualified researchers by Vivli (Burlington, MA) following review and approval of a research proposal, statistical analysis plan, and execution of a data sharing agreement. The data will be accessible for 12 months.
